# Internet of Things Based Blockchain for Temperature Monitoring and Counterfeit Pharmaceutical Prevention

**DOI:** 10.3390/s20143951

**Published:** 2020-07-16

**Authors:** Rajani Singh, Ashutosh Dhar Dwivedi, Gautam Srivastava

**Affiliations:** 1 Department of Digitalization, Copenhagen Business School, 2000 Copenhagen, Denmark; rs.digi@cbs.dk; 2 DTU Skylab, Technical University of Denmark, 2800 Kgs. Lyngby, Denmark; 3 DTU Compute, Technical University of Denmark, 2800 Kgs. Lyngby, Denmark; adhdw@dtu.dk; 4 Department of Mathematics and Computer Science, Brandon University, Brandon, MB R7A 6A9, Canada; 5 Research Centre for Interneural Computing, China Medical University, Taichung 40402, Taiwan

**Keywords:** scalability, blockchain, sensors, IoT, security, distributed network, supply chain, temperature monitoring

## Abstract

The top priority of today’s healthcare system is delivering medicine directly from the manufacturer to end-user. The pharmaceutical supply chain involves some level of commingling of a collection of stakeholders such as distributors, manufacturers, wholesalers, and customers. The biggest challenge associated with this supply chain is temperature monitoring as well as counterfeit drug prevention. Many drugs and vaccines remain viable within a specific range of temperatures. If exposed beyond this temperature range, the medicine no longer works as intended. In this paper, an Internet of Things (IoT) sensor-based blockchain framework is proposed that tracks and traces drugs as they pass slowly through the entire supply chain. On the one hand, these new technologies of blockchain and IoT sensors play an essential role in supply chain management. On the other hand, they also pose new challenges of security for resource-constrained IoT devices and blockchain scalability issues to handle this IoT sensor-based information. In this paper, our primary focus is on improving classic blockchain systems to make it suitable for IoT based supply chain management, and as a secondary focus, applying these new promising technologies to enable a viable smart healthcare ecosystem through a drug supply chain.

## 1. Introduction

**Figure 1:**
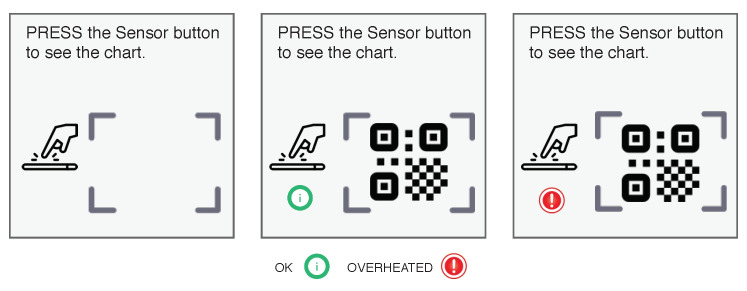
Temperature tracker sensor.

Transportation of pharmaceuticals (drugs, vaccines, supplies) from manufacturer to patient follows a stringent supply chain. The critical challenge of this supply chain management is safe transportation. The healthcare industry has an increasing number of counterfeit drugs on the market, and one in ten medical products are falsified in most developing countries. These medicines contain an incorrect ingredient or no active ingredient. Take for example, the current global pandemic of COVID-19. A British Broadcasting Channel (BBC) News investigation found fake drugs for sale in Africa, where counterfeiters are exploiting growing gaps in the market. The World Health Organization (WHO) said taking these drugs could have “serious side effects” [1]. The other key challenge is many pharmaceuticals are supposed to remain viable or effective only within a certain range of temperatures. If the temperature becomes too hot or cold, these medicines become ineffective, thus do not work as they are intended to. Being able to verify the temperature conditions and authenticity of medicines allow medical professional or patients to discard medicines that are no longer active or effective. When proper medication is found, the supply chain of that medication will need to be monitored closely both for the authenticity of medication as well as if any of the useful ingredients are temperature sensitive [2]. Patients, pharmacies, hospitals all rely on the medicinal supply chain and logistics companies that deliver heat-sensitive medicines, such as vaccines or insulin. During the transportation of these medicines over thousands of kilometres, a smart transportation box is required that maintains the temperature and records all changes into a pre-programmed sensor. The system of transporting and storing the vaccine at a certain temperature from manufacturer to the point of consumption is called *Cold Chain*. The essential elements of a cold chain are: *equipment for vaccine transport and storage* and *equipment to monitor the temperature of the vaccine*. Since trust among all participants and stakeholders in the supply chain or cold chain is required, incorporating any sort of blockchain technology should be considered a strong solution. Blockchain technology can store all sensor data and cannot be manipulated. Using blockchain, therefore, builds trust among a smart digital health ecosystem. All the important information about how drugs are entered and moved through the supply chain is available to everyone who is connected to the blockchain system. The sensor (see Figure 1) can collect the data—for example, the temperature inside the package while drugs are in transit from one place to another [3,4,5,6,7,8].

**Figure 2:**
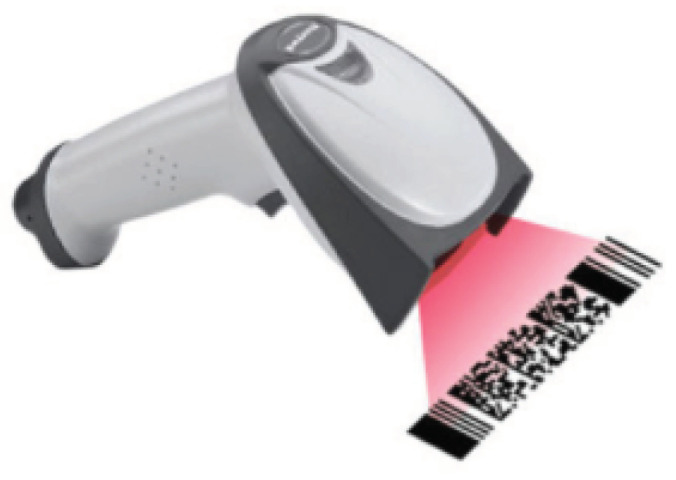
Quick response (QR) code reader.

The QR code stores all necessary information regarding sensor, temperature range, temperature threshold, owner information, etc. The QR code has two blank information cells (low temperature and high temperature). During transit, if the temperature changes beyond a given threshold for that particular drug, then it stores that temperature in the QR code blank cells. The sensor also shows a red light if the temperature has ever gone beyond the threshold and blinks green if the temperature has remained under the threshold. Once delivery is completed, sensor data can be transferred to the cloud using *QR code Readers* (see Figure 2) and the hash of the QR code is stored in a blockchain.

Nowadays, handheld smartphone devices are predominantly used as a QR code scanner and reader. However, the security of these sensor devices is another issue. Therefore, we need some digital signature scheme to confirm that the data is not modified by the attacker. The other issue with sensor networks is time synchronization of all sensors. Any form of sensor data fusion requires a synchronized clock. The problem of time synchronization in sensor networks was briefly described in the article [9]. To address the problem of time synchronization in sensor networks, the Berkeley algorithm [10] is one of the famous algorithms developed by Gusella and Zatti at the University of California, Berkeley.

One of the well-known drawbacks of blockchain technology is the scalability of the network. Scalability is defined as one of the most vital problems in blockchain technology and has been a prime focus in the blockchain community since its onset. The integration of IoT devices to the blockchain is challenging. IoT devices generate a large amount of data, and blockchains cannot handle them due to their low throughput or transaction execution rate. There are quite a few consensus algorithms available for blockchain that support high throughput; however, they require extremely high-performance for the network. In the proposed model, we used Raft consensus algorithms that provide high throughput, but Raft is only suitable for a small number of participants in the network. Increasing the nodes in the network decreases the efficiency and throughput of Raft due to low network scalability. The Raft algorithm expects very fast data transmission to provide high throughput. Therefore, we increase network performance by using the *bloXroute* [11] server concept. These bloXroute servers propagate the blocks very fast in the network. However, the bloXroute server propagates only encrypted blocks that prevent it from stopping the block propagation based on its content, and therefore node discrimination is not possible by servers. Please note that bloXroute is not a blockchain itself but it is a highly scalable distributed network only. Collecting encrypted data from resource-constrained devices, for example, the QR code scanner, is also a major issue. Currently, many applications like sensor networks or RFID are implemented on devices with very limited capabilities, and they require lightweight encryption. Many well-known standard algorithms, for example, AES, do not stand up to the basic requirements of constrained devices. This includes the need for minimal cost hardware implementation, minimal power usage, and minimal latency. In response to these problems, lightweight cryptographic primitives have been proposed in this model. These algorithms are usually smaller and faster for IoT based software implementation. Another major issue in need of addressing is the security of sensor devices with lightweight digital signature schemes. To know if the sensor data is coming from a reliable source, a digital signature can play an important role.

The rest of this paper is organized as follows. In Section 2, we summarize the recent related works connected to our study here. Next, our system is summarized in Section 3. We discuss several cryptographic techniques in Section 3.3. Our main evaluations occur in Section 4 and Section 5, where we give a performance evaluation and security analysis, respectively. The paper ends with some future directions and concluding remarks in Section 6.

## 2. Related Work

Malik et al. [12] proposed a three-layered framework for trust management known as TrustChain. TrustChain is shown as a blockchain technology-based application for supply chain, which is used to navigate trust issues that are linked to commodity quality. TrustChain makes use of a consortium blockchain that is used to monitor interactions with all participants on the supply chain participants. It also in dynamic fashion assigns both a reputation score and trust score solely based on interactions within the supply chain. Their intricate framework is also vital to give a reputation model that is both the asset and agent-based. The authors provide an in-depth security analysis focussing on threats to the reputation system. We can summarize the new notions of Trustchain stems as:

1 Based on several observations of supply chain events, the proposed model evaluates the product quality and entities trustworthiness.
2 Assigns the reputation scores to each participant and also product-specific score to the same participant in the supply chain.
3 It uses smart contracts for automated, secure, efficient and transparent calculation of the score.
4 The blockchain overhead in terms of throughput and latency is minimal compared to other supply chain blockchain models.

Caro et al. [13] presented a blockchain-based Agriculture-Food supply chain, named AgriBlockIoT. The proposed model integrates IoT devices with the supply chain and processes all the data in the supply chain with the help of IoT. AgriBlockIoT also integrates blockchain along with IoT to create auditable, transparent and immutable records used for the traceability of supply chain management. Authors used two different blockchains, Hyperledger Sawtooth and Ethereum. The proposed framework also developed a use case named as “from-farm-to-fork” and presented the pros and cons of their system by comparing and evaluating the latency, network usage and CPU performance.

Jamil et al. [14] proposed a blockchain-based framework to handle the pharmaceutical drugs supply chain. In the proposed, framework, a Hyperledger Fabric blockchain is used that is based on proof-of-concept consensus. The framework describes the implementation, design and performance of Hyperledger Fabric blockchain for smart hospitals. The blockchain system enables the pharmacists, nurses, patients and doctors to manage the healthcare ecosystem along with medical records. A smart contract is developed with solidity programming code and is also used along with a permission blockchain system. By utilizing this blockchain and smart contract-based system, the framework mitigates the issue of counterfeit drugs. However, they have not analyzed the supply chain management of biopharmaceutical drugs (for example, vaccines) that have special temperature and weather requirements, which is one of the most important problems nowadays.

Kapoor et al. [15] provided a comprehensive overview of the Pharmaceutical Supply Chain. They analyze the benefits of the supply chain and discussed strategic issues and challenges in the pharmaceutical supply chain.

Bishara [16] developed a framework for cold chain management of pharmaceutical drugs to manage the issues of risk assessment factors, quality management, distribution practices, and temperature monitoring. Developing a humidity and temperature monitoring system is essential to maintain the quality of biopharmaceutical drugs. Through our literature search, our efforts to find pertinent literature that dealt with the work we are proposing in this paper was limited.

When focussing on throughput, Gorenflo et al. introduced FastFabric, which is able to increase the throughput in Hyperledger significantly [17]. Furthermore, Stathakopoulou et al. also tackle increasing the low throughput in classic blockchain with work on Mir-BFT [18].

In our in-depth literature search, the work presented in this paper is the first look at such a framework in relation to drug transport protocols. The novelty of the work and contributions comes through the designed framework and use of secure components that, when used in tandem, provide a secure system for temperature-controlled pharmaceuticals to be transported through the supply chain.

### 2.1. Supply Chain Management

**Figure 3:**
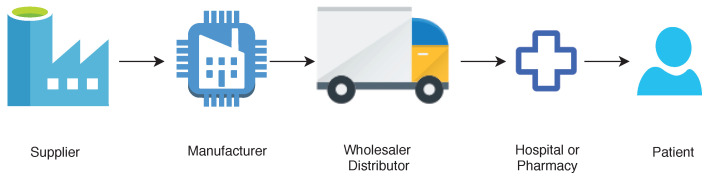
Supply chain management.

A supply chain is an interconnected network of nodes pertaining to organizations, individuals, technologies, resources involved in the manufacture and sale of the products (see Figure 3). A supply chain starts from the supplier who delivers the raw materials and ends with the end-user customer, for example, hospitals, patients, pharmacy shops. The supply chain takes care of movement and storage of produced medicines from source to the destination. A pharmaceutical drug supply chain system has the following important elements:

- **Manufacturers:** The manufacturer receives orders from wholesalers or distributors and ships the finally produced pharmaceutical drugs in large quantities to distributor warehouses.
- **Wholesalers:** The wholesaler propagates the process and distributes pharmaceutical drugs to pharmacies and hospitals. This saves time and effort of the manufacturer from the distribution of drugs.
- **Pharmacies:** Pharmacies and hospitals purchase the pharmaceutical drugs from wholesalers. The drugs received by pharmacies and hospitals are given or sold to end-users or patients.

It is important to note that some pharmaceutical drugs have a specific temperature range requirement, whether they are in storage or in transit. This temperature monitoring of pharmaceutical drugs will follow the *cold chain* process. A cold chain system consists of the following:

- **Cold storage:** A cooling system is required to maintain the required temperature of specific pharmaceutical drugs. It provides the facilities to store the drugs for a certain period of time: either drugs are waiting to ship to a distant market, or they are at pharmaceutical shops.
- **Cold transport:** The transportation companies can not invest a lot in making refrigerated vehicles to transport pharmaceutical products and therefore drug companies have to take this responsibility and to develop a kind of insulated box that maintains a certain temperature during transit.
- **Cold processing and distribution:** During the distribution of drugs, the cold chain has to ensure the sanitary conditions when consolidating and de-consolidating loads (crates, boxes, pallets) for distribution.

### 2.2. Drawbacks in Supply Chain

Some drawback of a supply chain can be mentioned as follows:

- During this process of the supply chain, drugs are transferred from the supplier to end-users, and counterfeit drugs can be merged at any level of the chain. Several pharmaceutical companies use holographic technologies to reduce the impact of counterfeit drugs. On the one hand, these types of packaging are expensive, and on the other hand, holograms can also eventually be cloned by counterfeit companies.
- During the process of the cold chain, for heat-sensitive medicines such as vaccines and insulin, it is important to track the temperature throughout the progress of the chain. Tracking the temperature of these vaccines all the way with trust among all parties in a supply chain is critical.
- Due to decentralized features and high-security, a blockchain could be the solution. However, the current blockchain cannot afford high throughput, and on the other hand, scalability of the blockchain network is low. Due to high bandwidth overhead and delays, the current blockchain models are not suitable for IoT devices.
- The data stored on the blockchain is public and therefore if organizations want to hide data from other organizations, then privacy might be an issue when using a blockchain.
- The security of sensor devices is the biggest challenge. It is important to ensure that the data is coming from reliable sensor devices and has not been modified.

### 2.3. Proposed Solution

An overview of our proposed solution can be given as follows:

- As pharmaceutical drugs travel through the supply chain and require trust among all parties involved, we incorporate blockchain technology. Due to the decentralized property of blockchain, it builds trust in the digital ecosystem.
- The proposed framework uses *Sensor* and *QR code scanner* that enable checksum of all temperature monitoring sensor data and packets QR code information. If sensor temperature goes beyond the threshold or if QR code data are changed on the packets, the system will not accept the packets anymore. The QR code used in the model are modern clone-proof and secured.
- We build a scalable distributed network with the help of *bloXroute* [11] that provides high network scalability and uses the high throughput Raft consensus algorithm, which makes our system suitable for IoT devices. The blockchain stores all pieces of information produced from sensors and QR code readers.
- In case the group of organizations wants to keep information private from other organizations, the proposed model uses Hyperledger. Hyperledger uses separate channels where the transactions (ledger) of one channel is confidential and hidden from another channel.
- In the proposed system, it is easy to prove at any time that drug packets or sensor data have not been manipulated. We used digital signature schemes for the security of sensor data.

## 3. Our System

We break down our system into the following two major contributions:

- Building a scalable and high throughput blockchain distribution network (BDN) with the bloXroute server and Raft consensus algorithm that is suitable for IoT devices with high throughput.
- Building an Internet of Things (IoT) and blockchain-based supply chain management for pharmaceutical drugs.

### 3.1. Blockchain Network

**Figure 4:**
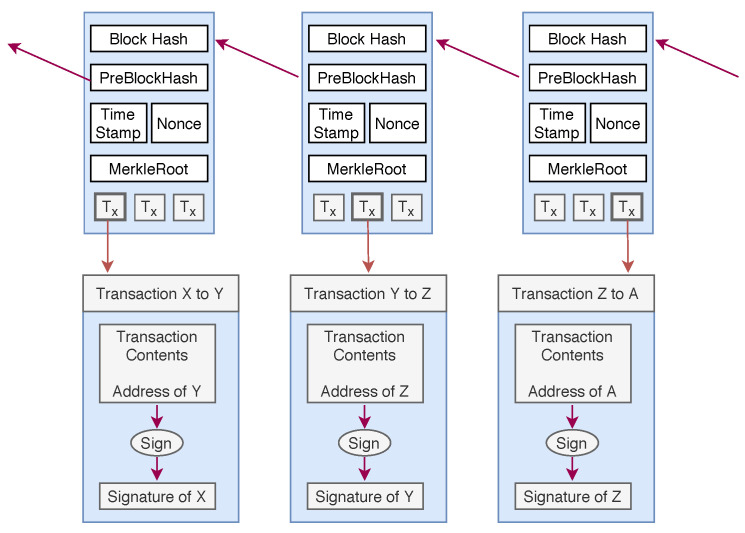
Structure of a block in PoW based blockchain.

Initially, the concept of blockchain was introduced by Satoshi Nakamoto [19] for applications in the domain of cryptocurrency and later it become popular in several applications of other fields [20,21,22,23,24,25]. Blockchains can be simplified in description to a distributed ledger, which can be append-only. There is no central authority who controls the data. All nodes in the network come to a consensus to verify a set of data that is saved in a block. Once a block of data is verified, it is appended to a chain of blocks. These blocks are connected by hash, and due to the use of hash and cryptographic signatures, it is almost very tough to alter data. This data chain is also called a blockchain, and the copies of this blockchain are stored at many places and therefore easy to access by anyone in the world. Each block (see Figure 4) in the network consists of Transactions and other information, such as Block Header and Merkle Root. Each block in the blockchain is identified by the hash, which is saved in the header. The first block of the chain is called *Genesis*. In order to integrate the blockchain with IoT devices, our primary goal is to build a scalable distributed network that provides high throughput.

#### 3.1.1. Throughput Scalability

Generally, the throughput of a protocol in the blockchain system is represented by a number of transactions per second (TPS) or number of blocks added into the blockchain per second. Throughput of the current Ethereum public blockchain is 10–30 TPS approximately while Bitcoin blockchain throughput is 3–7 TPS. Therefore, throughput produced by Ethereum or Bitcoin is not adequate for the applications where a higher rate of throughput is required. A private blockchain-based supply chain network requires at least 1000 TPS for the entire system. Raft consensus algorithm can be best suited for our requirement due to its high throughput, but Raft consensus algorithms are only suitable for small network due to its high-performance network requirement (see Table 1). Throughput [26] of a Bitcoin-based blockchain system depends on the following two parameters:

1. Block size: It represents the total number of transactions in a block and is denoted by *B*.
2. Inter-block time interval: It is the time taken by miners in mining a new block and denoted by $t_{B}$.

For example, throughput for a block containing 2000 transactions with the average time 600 s is 3.33 TPS. Therefore, it is necessary to either decrease the Inter-block time interval of $t_{B}$ or increase the block size *B*, to boost the throughput. Later we will discuss that these parameters cannot be changed arbitrarily.

### Block Size (B):

**Figure 5:**
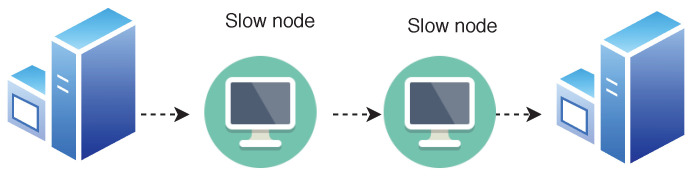
Peer-to-peer network.

Due to the limited P2P propagation capacity, it is practically infeasible to increase the block size. The current processor maximum throughput is 1000 TPS while input-output disk can support 10 times of processors TPS. After mining a new block, the miner propagates the block for verification across all the network. In general, a peer-to-peer network consists of several nodes, and because of the slowest computer in the P2P network, it might be possible that the propagation speed is slow (see Figure 5). Therefore, by increasing the block size, the time required for a block to propagate [29] will also increase. Now, let us discuss what will happen when propagation time for a block is increased.

### Increasing the Propagation Time of Block $\left(t_{B}\right)$:

**Figure 6:**
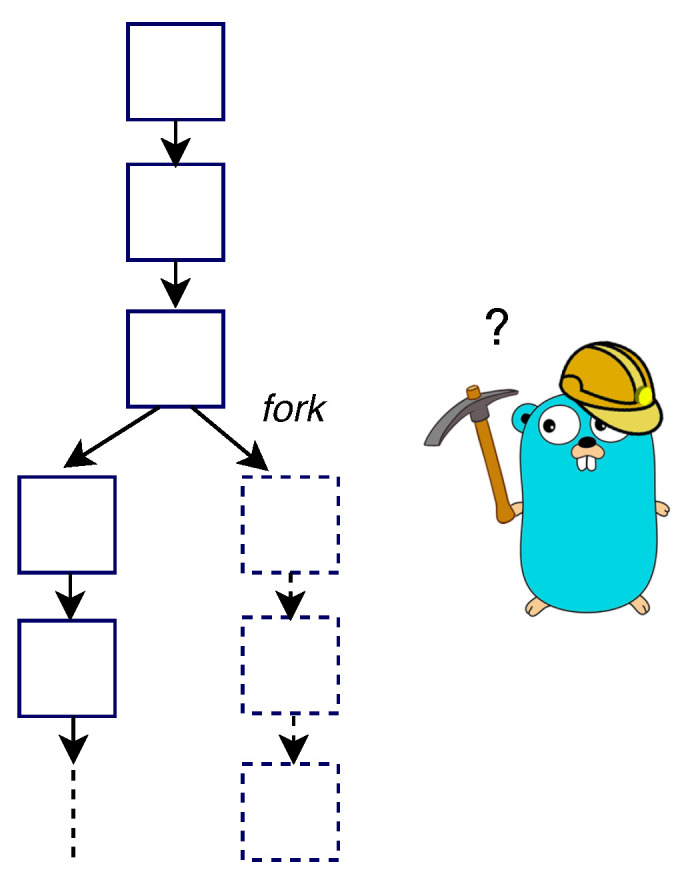
Fork in blockchain.

It is also possible that the *fork* may appear because of increments in the time during the block propagation. This will result in a change in the protocol. If a fork occurs, miners will not mine the new block on top of the recent block; instead, they will mine on the previous block. This is because the miners have not yet got the latest mined block (see Figure 6). Hence, the block propagates time that is needed by a new block to reach it throughout the whole blockchain network, which opens the opportunity window where forks may occur. In other words, the longer the propagation time, the higher the probability that the fork will occur. For example, for a block, with 600 being its block propagation time, i.e., ($t_{B}=600)$ and $t_{network}$ being the time needed by a block to reach throughout the whole network. The probability of the fork occurring is [30]:

(1) $$P\left(\mathrm{fork}|t_{B}=600\right)=1-\epsilon^{\frac{{}^{-t}network}{600}}$$

Simplify Equation (1), to get the probability of the fork occurring as P(fork)=1.95% for block propagation time $t_{network}=11.6$ s. If the $t_{network}=126$ s, then P(fork)=17.58%, which is unacceptable or infeasible for real world usability.

### Shorting the Block Propagation Time $\left(t_{B}\right)$:

Now, consider the second alternative of decreasing the block propagation time $\left(t_{B}\right)$. Decreasing $\left(t_{B}\right)$ might stop multiple nodes from participating in the Blockchain network. To allow a maximum of nodes to participate actively in the network, the propagation time of the blockchain network must be shorter than the threshold block propagation time $\left(t_{B}\right)$, as shown in Equation (2).

(2) $$t_{network}<t_{B}$$

Therefore, the ratio between the network propagation time $t_{network}$ and block propagation time $\left(t_{B}\right)$ needs to be appropriately maintained. Finally, to increase the throughput of the blockchain, inter-block time interval $\left(t_{B}\right)$ can be reduced, for that our requirement is to decrease the $t_{network}$. Hence, we lowered the inter-block time interval $\left(t_{network}\right)$ for the whole blockchain network in our proposed model.

#### 3.1.2. Blockchain Scalability Solution Using BDN

**Figure 7:**
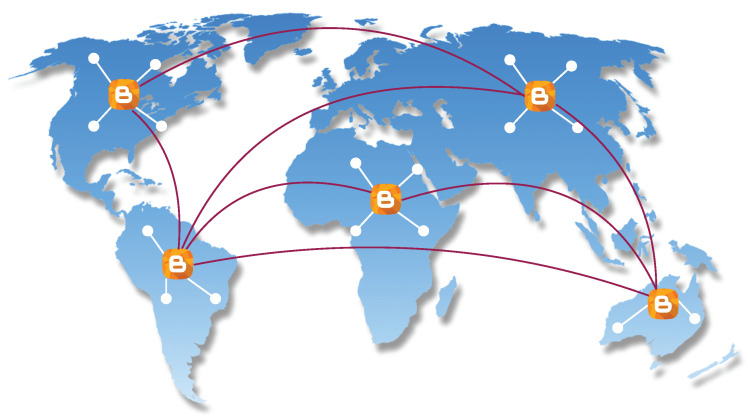
Distributed blockchain servers connected with peer networks.

Our primary goal is to increase the throughput of the system, that is, scaling the system with 1000 TPS but instead of a small network (e.g., Raft), we need this throughput for a large network. Applying Raft consensus is only possible when we have a highly scalable distributed network. Therefore, we need a highly scalable network to increase throughput. We use the *bloXroute* server concept, which solves the scalability bottleneck at its core at layer zero, underneath the blockchain at the network layer. Our network is a blockchain distribution network (BDN)—a global network of servers (see Figure 7) optimized for quickly sending blockchain data. These BDN servers use advanced network techniques—when a blockchain server receives a packet of data, it immediately streams this data to the rest of the network allowing the blockchain server to propagate data up to 100 times faster. By removing the networking bottleneck, the blockchain server solves the scalability problem for the blockchain. In our system, we use distributed high-capacity servers. The system consists of two levels of topology network:

- Blockchain servers that are both low-latency and high capacity servers and are optimized to propagate transactions and blocks for multiple blockchain systems quickly. They work like cluster servers connected with other clusters. These blockchain servers decrease network overhead and delay. Note that these servers do not act as a central server and manage other small nodes, but the purpose of introducing them is to increase the propagation speed of blocks.
- Peer networks that are P2P networks of computer or mobile nodes that use blockchain servers to propagate transactions and blocks, while they also audit the behaviour of the blockchain server. These peer networks use a specific consensus algorithm. These networks are grouped in the form of clusters and each cluster has one blockchain server that propagates transactions and blocks on behalf of small nodes also called peer.

**Figure 8:**
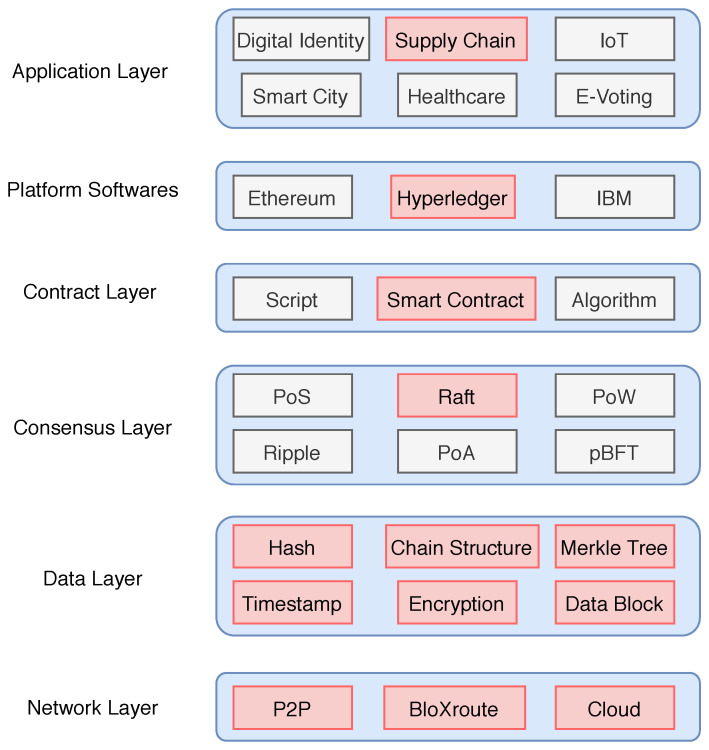
Blockchain architecture.

Different peers send encrypted blocks to blockchain servers. Peers can send the encrypted block to another peer instead of directly sending the block to server. Because of this setting, the blockchain server cannot cheat any particular node or cluster. The blockchain server blindly serves the nodes in the network without knowing the content inside the encrypted block. Due to fast propagation speed, these blockchain servers forward blocks quickly to other networks for the verification without any network delay. To audit the behaviour of any blockchain server, the peers in the network can send test blocks to the blockchain server and validates if their peers quickly receive them. The complete blockchain architecture for the proposed framework is shown in Figure 8.

#### 3.1.3. Consensus Algorithm

There are several consensus algorithms for blockchains and a few important consensus algorithms were presented in Table 1. We note here that *transaction per second* is not very high for PoS or PoW based algorithms. Raft based consensus algorithms have high throughput but low network scalability, and therefore, it has extremely high-performance requirements for the network.

However, in our model, we increase network scalability by using high bandwidth distributed blockchain servers in the network just to speed up the block propagation speed in the network, and therefore a Raft consensus algorithm is suitable for our needs. The ability of a distributed network to reach consensus in the presence of malicious nodes is called Byzantine Fault Tolerance (BFT). These malicious nodes send the wrong information to the network. To avoid any catastrophic system failures due to these faulty nodes, BFT reduces the faulty node influence in the network. The main objective of BFT is to reduce the influence of these faulty nodes in order to protect the system against such catastrophic system failures. In distributed systems, replication is mainly used to provide fault tolerance. However, the modular architecture of the hyper ledger allows both BFT (byzantine fault-tolerant) ordering or CFT (crash fault-tolerant). Raft has been implemented in various modern distributed networks along with several blockchain platforms. Hyperledger Fabric is a famous blockchain that uses Raft.

**Figure 9:**
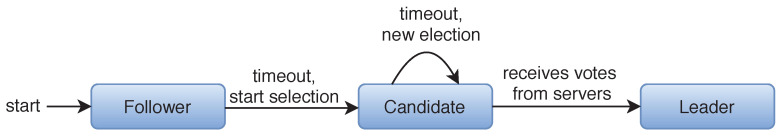
State transitions of Raft members.

Raft is based on the leader and follower scenario where a leader node is selected and responsible for managing the cluster. All the nodes in the network have three states: Leader, Follower or Candidate. Only one node can be a leader at a time. A leader node handles all the communication from the client and follower node response to incoming RPCs. The follower waits for an incoming message from the leader, and if not received, the follower assumes that the leader is dead (see Figure 9). A new leader is chosen on the basis of a new election.

#### 3.1.4. Distributed Ledger Software

The same blockchain ledger can be used by many organizations and in cases where organizational groups want to hide data from other groups, in such cases, we need separate channels to store the data of different groups of organizations. Creating separate channels causes additional overhead (maintaining separate chains of blocks). *Hyperledger Fabric* offers the ability to create such kinds of separate channels or private data collections called chaincodes. Hyperledger Fabric is an open-source project hosted by the Linux Foundation, which provides a collaborative environment for a permissioned blockchain. Fabric is the first truly extensible blockchain that allows various components, such as consensus algorithm and membership services to plug and play. Fabric currently powers more than 400 prototypes and proofs-of-concept of distributed ledger technology and several production systems, across different industries and use cases. Hyperledger Fabric is the best choice for the B2B supply chain services that use a Raft consensus algorithm for higher throughput.

#### 3.1.5. Distributed Database Network

**Figure 10:**
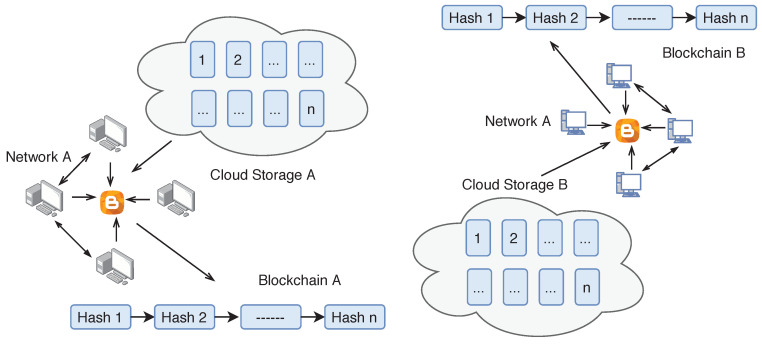
Cloud network.

The blockchain only stores the event of the supply chain while actual data is stored on the distributed cloud. The data stored on the cloud is in the form of identical blocks associated with a unique block number (see Figure 10). These clouds are connected with the peer network as well as bloXroute servers. The hash of the data is calculated using the Merkle Tree structure, and each block has a different Merkle Root. The Merkle tree is stored on the blockchain. Any change in the data is easily traceable using blockchain, and therefore the proposed framework does not require complete third party trust.

As an alternative to cloud storage, one can also use the decentralized cloud storage network such as *Storj* [31]. Working of Storj is explained below:

- **Data storage:** To use the Storj network to save the data, first, the client or user breaks the data into multiple data chunks, which is called sharding. After sharding, these data chunks are distributed to different peers across the decentralized cloud storage network. When these chunks are being sent and stored into different peers, metadata is created containing all the information related to data retrieval, i.e., where to find the data chunks again.
- **Data Retrieval:** To get or retrieve any data from the Storj network, the client or user will first reference the metadata to check the location of previously stored data chunks. Then, the original data is retrieved by simply reassembling all the data chunks on the client’s system.
- **Data Maintenance:** If certain data have multiple copies and this redundancy went below a certain threshold level, the necessary data for the missing data chunks are reconstructed and replaced.
- **Payment** There is some cost associated with using this cloud storage, which has to be paid in units of usage.

### 3.2. Blockchain and IoT Based Supply Chain Management

**Figure 11:**
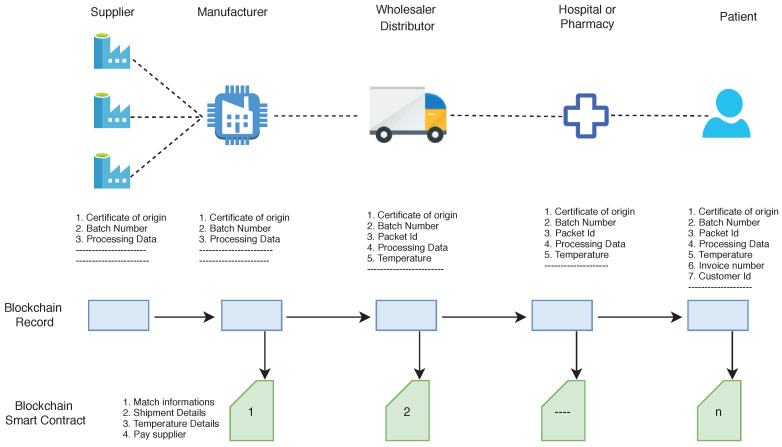
Blockchain based supply chain of pharmaceutical products.

In a supply chain of pharmaceutical products, the wholesaler purchases the product from the manufacturer. Depending on the demand, it is also possible that the wholesaler purchases the products from another wholesaler. After repackaging the products, the wholesaler distributes them to the pharmacies or hospitals. When the product is transferred from one entity to another entity or one level to another level of the supply chain, the asset is changed, and we need to assign a new identity to the products. In this process of packaging, counterfeit products can be merged at any level. The proposed framework (see Figure 11) is to create a blockchain and IoT based system that records and timestamps the transfer of goods at each point in the supply chain management. As the products and vaccines travel through the supply chain, every transaction of pharmaceutical products and vaccines should be timestamped with the current supply chain owner. The ledger stored in the blockchain is used to ensure the security and safety of the products. The patients, hospitals or pharmacy, anyone can access the full history of the products they received. The other key challenge is that many products like vaccines are viable with a certain temperature range, and in case there would be a break in the cold chain, the medicine becomes no longer active. Several solutions exist in the literature to prevent these risks, such as the use of refrigerated vehicles or use of thermoboxes/isothermal boxes. These isothermal boxes contain eutectic plates that allow constant cold diffusion to maintain a uniform temperature level. However, monitoring of temperature is still a big challenge.

We build a blockchain-based framework with the following components:

- **QR code scanner:** A QR code scanner or smartphone with QR code scanning application is required to read the information of packets and to store them in the blockchain.
- **Asset creation:** Once a product is first entered into the supply chain, a new asset is created. All product information is stored in the QR code. With the help of a QR code scanner, this information is entered into the blockchain.
- **Sensor:** A sensor is required to assemble with cold chain products that require temperature monitoring. These sensors are mainly required when products are in transit, and once a next level entity in the supply chain receives the product, the sensor QR code is scanned, and if overheated, the entity can deny acceptance.
- **Asset transfer:** If the asset is transferred from one supplier to another supplier or one entity to another entity in the supply chain, the blockchain stores the change in information.
- **View scanned product:** All participants can view the product details in the supply chain who are registered with the mobile phone application. Once a user scans the QR code from the drug packets or sensors associated with the packets, the user gets all information about the supply chain or cold chain.

**Figure 12:**
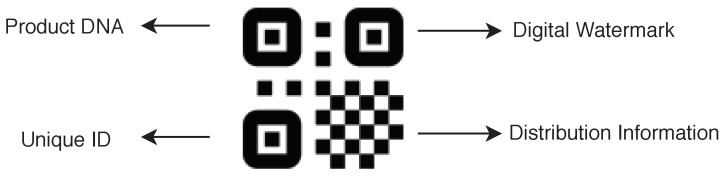
Clone proof QR code.

To make the supply chain secure, a QR code (see Figure 12) should also be clone-proof. Traditional QR codes are less secure and can be copied quite easily. In the last few years, several companies have introduced new QR codes that have more security features. In the proposed model, we recommend the use of digital watermark based clone proof QR codes (see [32]), known as AR Codes. The QR code provided by them is clone-proof and provides a solution for trace and track and anti-counterfeiting. The QR code can be scanned using a normal mobile phone by using a mobile application or web application.

**Figure 13:**
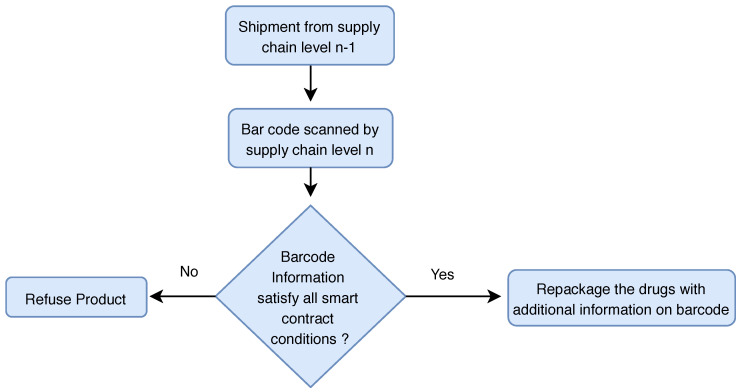
Information flow in the supply chain.

Once the manufacturer produces the final product in a large extent and bundles them in packages, a QR code is created, which adds batch number and certificate of origin to these packages. Generally, in the case of self-certification, the manufacturer issues the electronic certificate of origin, and this certificate is also added to the blockchain. However, if third-party certification is required, first the certificate must be signed by the manufacturer and then countersigned by a local issuing body, such as a chamber of commerce (board of trade) or customs authority. All products that are packed under one manufacturer should have the same batch number. During the transit of packages in the supply chain, once the package is shipped from manufacturer to distributor or from distributor to retailer, it is scanned through a QR code reader. If the QR code information does not match the condition, for example, overheated temperature of vaccines or batch number modified, then the receiver does not accept the package (see Figure 13).

After receiving and authenticating the package from the manufacturer, the distributor repackages the individual products from the package and adds a serial number to each individual product. At each level, information is added to the QR code, but previous information cannot be removed that is already stored. For example, if the product comes from the manufacturer ID: xyz and distributor ID: ABC then the retailer can add his ID (say 123 but cannot remove previous IDs from QR code information, giving a resulting QR code concertation of xyzABC123.

### 3.3. Cryptographic Techniques Used in the Network

In the proposed model, we have used several cryptographic techniques for privacy and security of the data. For the purpose of encryption, we used a lightweight encryption algorithm suitable for IoT devices along with Elliptic Curve Diffie–Hellman Key Exchange and Digital Ring Signature.

#### 3.3.1. Lightweight Encryption for the Network

The purpose of encryption is mainly to provide confidentiality. In the proposed model, encryption is not used to provide confidentiality against supply chain users, but it is required for bloXroute servers. To achieve neutrality and auditing of the servers, bloXroute designers suggested the use of encrypted blocks, which prevents servers from stopping the blocks based on its content. In general, any blockchain system, especially cryptocurrency, uses public-key cryptosystems for transactions and wallets but in our model we are also using IoT devices to read and store QR/AR codes. Therefore, very lightweight encryption schemes are needed. Several encryption schemes [33,34,35,36,37] were presented in cryptography competitions for encryption algorithms [38,39]. With the growing number of resource-constrained devices in IoT, it becomes challenging to choose the encryption scheme based on system requirements. A blockchain network consists of several resource-constrained nodes. The IoT nodes have low storage capacity, limited processing speed and low communication bandwidth. Some of the major constraints are described below:

- **Memory Limitation:** A sensor device may have very small storage and memory. These sensors generally use 4K bytes of SRAM, 128K bytes of programmable flash memory and an 8-bit register.
- **Energy constraints:** Energy limitation is the biggest issue of small IoT devices. Energy consumption is divided into the following categories: energy for communication and energy for the computation. The more instructions executed, the more battery is consumed.

**Figure 14:**
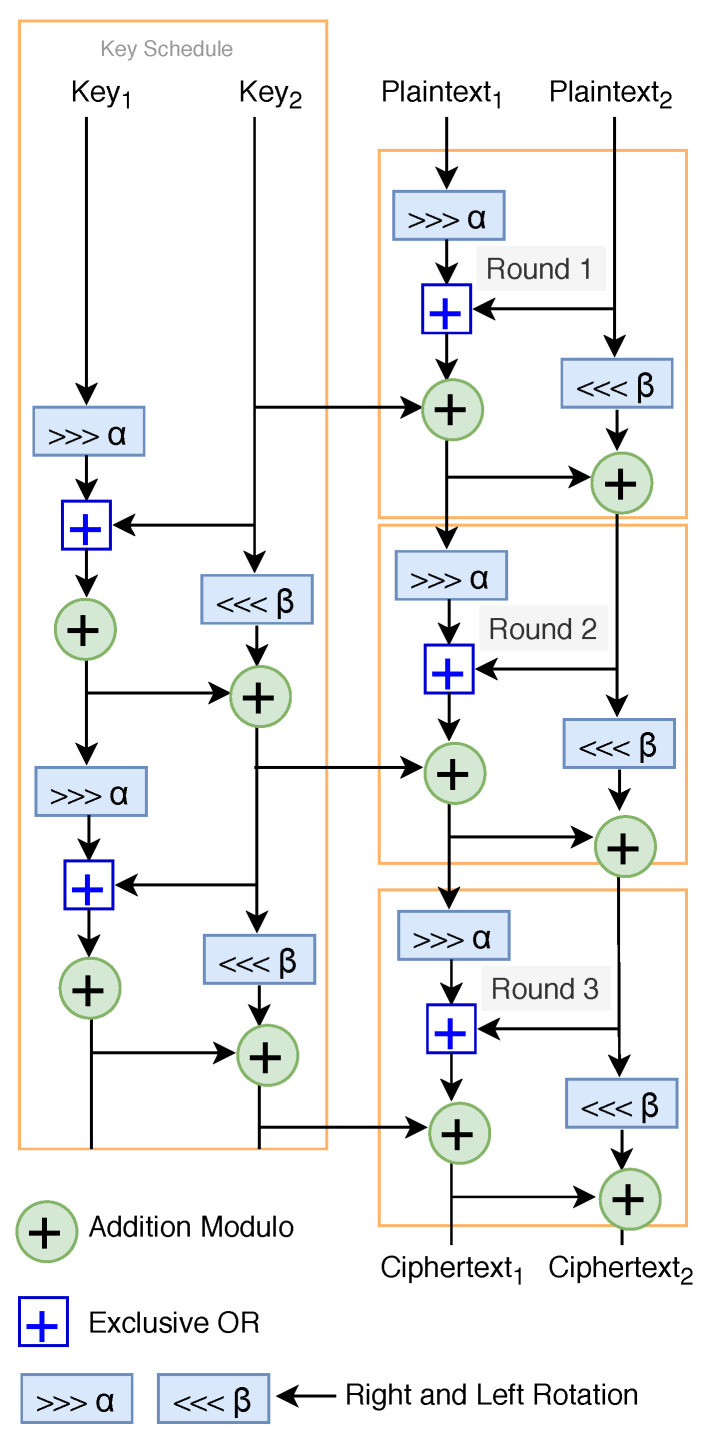
Lightweight encryption algorithm (SPECK) suitable for IoT devices.

AES is the standard encryption algorithm used nowadays with many applications [40]. However, AES is not well suited for lightweight encryption schemes. It uses heavy encryption components like S-box and, therefore, slows performance on IoT devices. The suitable encryption algorithms for IoT devices are in the ARX family. The ARX encryption family uses three basic operations, namely, modular addition, bitwise rotation, and exclusive-OR, and therefore, it is very well suited to perform on devices with low capacity, for example, SPECK [41], LEA [42,43]. SPECK was designed by the National Security Agency (NSA) researchers in June 2013. The cipher encrypt the plaintext (see Figure 14) with a fixed block size rather than bit by bit. The algorithm is safe against a different type of attack for the full round. Based on the security, throughput and energy consumption performance, we chose the SPECK encryption scheme for the proposed model. The performance evaluation of SPECK is presented in Section 4.2.

#### 3.3.2. The Elliptic Curve Diffie–Hellman Key Exchange

In symmetric-key encryption, the same session key is used to encrypt and decrypt the data and therefore choosing an efficient as well as lightweight key exchange algorithm is another challenge. In public-key cryptography there are several cryptosystems such as RSA, Elgamal and Diffie–Hellman. RSA is mainly based on the integer factorization while Elgamal and Diffie–Hellman are based on discrete logarithms. The Diffie-Hellman key exchange algorithm works in any group in which both the discrete logarithm problem is hard and exponentiation is efficient; for example, a group of points defined by an elliptic curve over a finite field $Z_{p}$. Here, we use another variant of the Diffie–Hellman secret key exchange algorithm that is based on the Elliptical curve.

The main advantage of using Elliptical curve for Diffie-Hellman key exchange is that it provides similar security as the above cryptosystem but with smaller key size, which makes the algorithm most efficient. The Elliptic curve Diffie–Hellman uses a 160-bit key to provide the security level of 80 bit (see Table 2). This means if a computer wants to break the 160-bit key system using exhaustive search, then it requires $2^{80}$ computational power. An elliptic curve over a finite field $Z_{p}$ (for a large prime number p>3) is a set consist of all points $\left(x,y\right)\in Z_{p}$ that fufill the Weierstrass equation $y^{2}≅x^{3}+ax+b\;\mathrm{mod}\;p$ and the non-singularity condition $4a^{3}+27b^{2}\neq 0\;\mathrm{mod}\;p$, where $a,b\in Z_{p}$ together with a special point known as *the imaginary point at infinity* denoted by *O*. Note that the points on an elliptic curve together with *O* have cyclic subgroups. Moreover, under certain conditions, all points on an elliptic curve form a cyclic group (see [44]). We denote the order of the elliptic curve by *n*. For implementing the Diffie–Hellman algorithm, we denote an elliptic curve by *E* and a point *P* on *E* that is a primitive element by $P=(X_{p},Y_{p})$. Note that the Diffie–Hellman secret key exchange algorithm is based on the discrete log problem assumption. In an elliptic curve for given *E* and point *P*, the discrete log problem can be defined as:

Findintegerd∈(0,⋯n)suchthatP+P+⋯+P=dP=T,

where *T* is another point on *E*.

**Figure 15:**
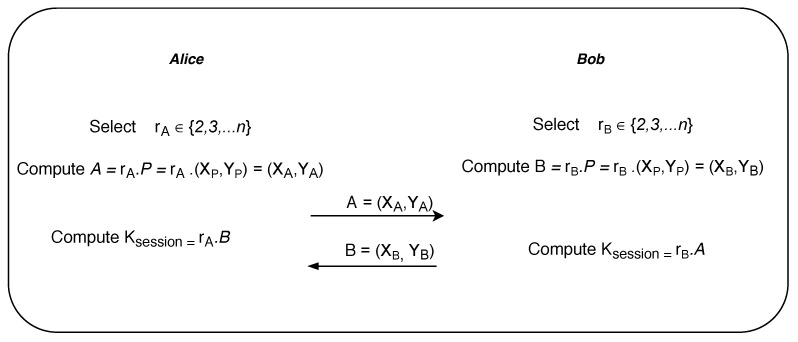
Elliptic curve Diffie–Hellman secret key exchange.

To explain the inner workings of the Elliptic curve Diffie-Hellman secret key exchange algorithm, we consider two popular fictional characters in cryptography, namely Alice and Bob (see Figure 15). Suppose Alice and Bob want to exchange the secret key or session key over the insecure public network. Alice randomly chooses a secret number $r_{A}\in \left(2,\cdots ,n\right)$ from the set of points on elliptic curve which is private to her. Similarly, Bob also randomly chooses $r_{B}\in \left(2,\cdots ,n\right)$ from the same set which is private to him. Now both compute the public parameters *A* and *B*, which is another point on the elliptic curve, by scaling the primitive point *P* by their secret numbers $r_{A}$ and $r_{B}$ respectively. Alice sends *A* to Bob while Bob sends *B* to Alice. Finally, Alice and Bob compute the unique session key $K_{\mathrm{session}}$ by again rescaling the received points *A* and *B* with their secret numbers $r_{A}$ and $r_{B}$ respectively.

#### 3.3.3. Digital Ring Signature

**Figure 16:**
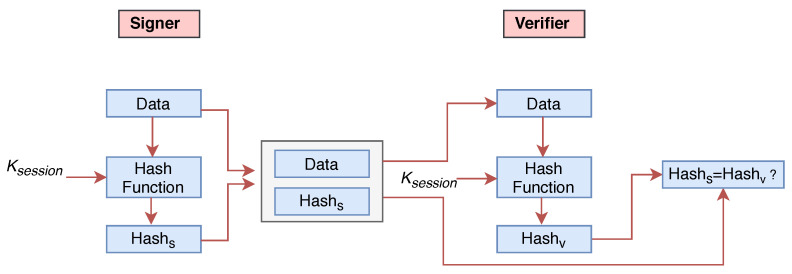
Digital signature using Hash.

The purpose of a digital signature is to provide authenticity, integrity and non-repudiation of data. These three features play an important role in supply chain management. In public-key cryptosystems, separate key pairs are used to sign and verify the data. The signer uses a private key to create a digital signature and receiver uses the signer’s public key to verify the digital signature. Another good alternative is to create a digital signature by using a hash function (see Figure 16). The digital ring signature provides two important security properties: Signers anonymity and Signers correctness.

The signer uses session key $K_{\mathrm{session}}$ (key is already exchanged using ECDH over an insecure channel) and generates the hash value of the document. Similarly, the receiver also generates the hash value of the received copy with the same session key $K_{\mathrm{session}}$. If both hash values match, then the data is not modified.

**Figure 17:**
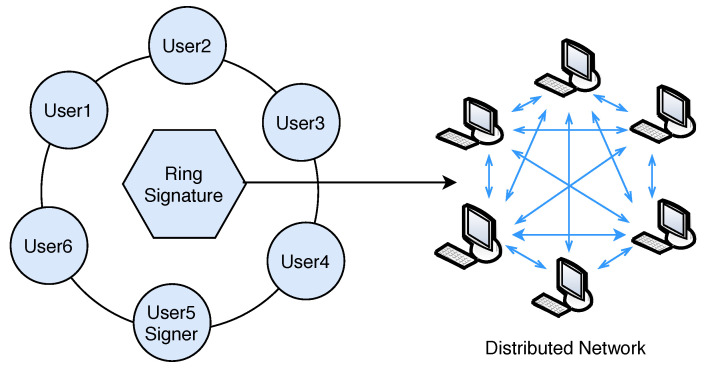
Lightweight ring signature.

Privacy in the blockchain is a major issue where transactions or users identity should be untraceable and un-linkable. For the purpose of anonymity, the framework uses a lightweight digital ring signature [45]. The basic idea behind the ring signature (see Figure 17) is mixing the users signature with other members, and no one in the network can identify which member signed the message. Ring signature was originally proposed by Rivest in 2001 [46].

## 4. Performance Evaluation

In this section, we present the performance evaluation of the scalable blockchain network and lightweight encryption scheme proposed in our model.

### 4.1. Performance Evaluation of the Network

In this scenario, we use the slowest consensus algorithm PoW based Bitcoin network with our scalable network where the network consist of approximately 10,000 nodes and each node has 8 to 12 peers with latency 110 milliseconds [26]. At the 50th percentile, the node upload rate is 56 Mbps ($bw_{50\mathrm{th}}$ = 56 Mbps. Let *B* be the size of the block, the time required to transmit single 1 MB block to single peer ($t_{hop}$) is:

(3) $$t_{hop}=\frac{B}{bw_{50\mathrm{th}}}=\frac{1\;\mathrm{MB}}{56\;\mathrm{Mbs}}=0.143$$

Due to limited bandwidth speed, nodes have to propagate blocks to their peers in sequential form. If a single block takes $t_{hop}$ time to propagate then 8 to 12 blocks can propagate in $8t_{hop}$ and $12t_{hop}$ time respectively. This is just because peer nodes propagate the received block after $t_{hop}$ has passed. However, due to the very high speed distributed blockchain servers for each peer cluster, the speed of the propagation can be increased significantly, and therefore, we can propagate the blocks in parallel form.

**Figure 18:**
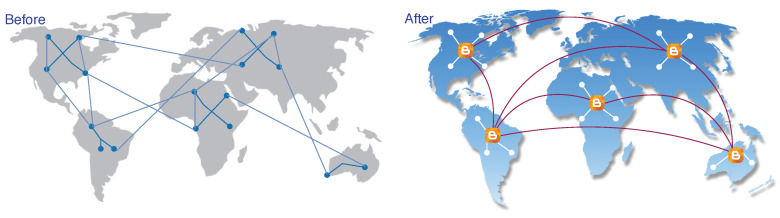
Blockchain distribution network.

In Figure 18, the first picture shows when peers propagate the blocks without bloXroute server, and therefore the propagation speed is slow while in the second picture framework uses the bloXroute server to speed-up propagation of blocks, and therefore it solves the network scalability issue. In the second case, eight blocks can be propagated in $t_{hop}$ time only and therefore if the normal throughput of Raft is 2000 TPS then after using the bloXroute servers, the throughput becomes 16,000 TPS. Note that, we only use high-speed servers above all networks just to decrease the propagation time of blocks, and we are not converting the whole network in the form of distributed central servers.

### 4.2. Performance Evaluation of the Encryption Scheme

**Figure 19:**
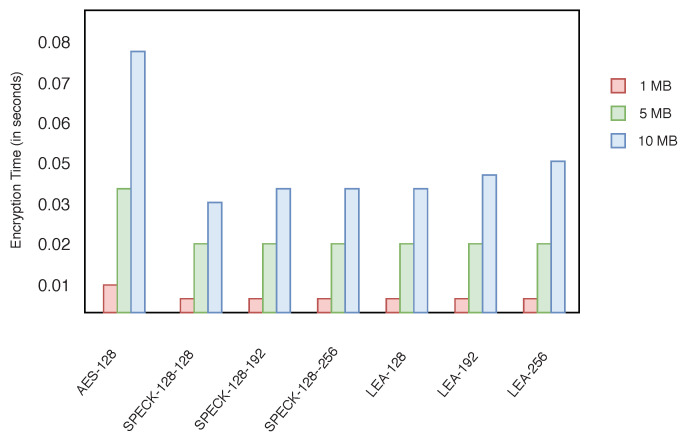
Average block cipher encryption time (seconds).

The performance of SPECK is always better in terms of encryption time (see Figure 19) and energy consumption (Table 3). The energy consumption is also tested for *Basic*—128-bit key and *Advanced*—192-bit key. The larger key size provides high security, but energy consumption is high.

Based on performance analysis and security margin against various attacks [47,48], in our proposed model, we chose SPECK (Figure 14) cipher, which is a lightweight encryption algorithm from the ARX family.

## 5. Security Analysis of Blockchain Distribution Network

In any IoT based model, few security requirements are needed to be addressed by the model designers. For example, authorization guarantees that only the allowed users are using the system while the data confidentiality guarantees that only these authorized users can access the system data. Data integrity guarantees that the data is not being tempered. Quick response code (QR code) security guarantees that the codes cannot be cloned and tempered by any defaulter. There are some other security requirements such as privacy, anonymity, and scalability that make the system model better and therefore, can also be used for the more general case. In Table 4, we present how we address these security requirements.

We considered a few attacks that could be possible in this model and find a security margin against them in our model. Security is evaluated in our blockchain-based model based on the three categories: Adversaries can be a trader who change the sensor data, blockchain server or a malicious node from the blockchain network. These adversaries can create false transactions, delete or change information, replicate fake nodes, discard transactions or modify watermarks. P2P nodes perform auditing in the server, and therefore, without discrimination, the server can propagate all blocks to gateways. However, except sensor tempering, we mainly focus on blockchain network security because once the data is stored in the blockchain, it enjoys the security properties of a distributed network.

### 5.1. Sensor Tampering

In the proposed model, an attacker or dishonest trader can change the data from the sensor device. In such cases, the sensor will provide an acceptable temperature reading. To enhance the property of authentication, integrity and non-repudiation, a digital signature has been used. Any changes in the data can be easily identified by end-users.

### 5.2. Packet Stuffing Attack

A dishonest trader may add counterfeit drugs during repackaging. However, in the proposed model, each small packet also includes a QR code along with the big boxes (e.g., case, pallet). The small packets under a large box (outer package) have the same batch number. Therefore, the batch id added by the manufacturer to each small packet during the first packaging cannot be modified later. However, even after that, if a trader adds counterfeit drugs, it is easy to trace it later due to digital signature and blockchain traceable property.

### 5.3. Storage Attack

It is also possible that an adversary can make a cloud storage attack and try to remove data, or modify data from the storage. However, in the proposed model, a hash is generated for each data inside the block, and a Merkle root is created for each block and stored on the blockchain. Therefore, any changes in the cloud data can be easily verified.

### 5.4. Encrypted Blocks

In the proposed model, each transaction is first encrypted by using a lightweight encryption scheme and then stored into blocks. Encryption of the transactions is necessary to prevent any discrimination based on contents like timestamp, transactions, and other attributes. When the transaction is transmitted through the blockchain server or gateway, the encryption key is revealed.

### 5.5. Indirect Relay

In our model, nodes do not propagate blocks directly to the blockchain server. Instead, the node propagates to the neighbour nodes, and then the neighbour node propagates it further and at some stage to the blockchain server through the other peers. In this way, we ensure that none of the individual nodes are being prevented from the propagation of the blocks.

### 5.6. Test Block

Our scheme enables the nodes to check directly whether the blockchain distributed server is working properly or not. Also, it prevents the blockchain distributed server from making any biased decision based on individual block contents. Any node can continuously monitor the functionality of the blockchain distributed server by sending a test block directly to it.

### 5.7. Dropping Attack

It could also be possible that the attacker controls some blockchain server so that the servers will be blocked and unable to perform in the network. To address this problem, we propose that peer networks can elect other nodes as their blockchain server. It is infeasible for the attacker to attack all the blockchain server at the same time. Therefore, our model is secure from this kind of attack.

### 5.8. Denial of Service (DoS) Attack

In a Denial of Service (DoS) attack, the attacker aims to make a machine or accessing the network unavailable to its intended or authentic users. An attacker can do this by temporarily or indefinitely disrupting services of nodes connected to the Internet. For example, an attacker can launch many fake transactions to block the traffic. However, in our model, we are using a permissioned blockchain since in such a blockchain, it is not allowed that a random user joins the network without validating the proof of authenticity. Moreover, if a node is detected for doing a malicious activity once, it is directly blocked by the peer network. This technique makes our model secure against such an attack.

## 6. Conclusions

In this paper, we present a novel, blockchain-IoT based supply chain management system to mitigate the problem of counterfeit drugs and to properly monitor the cold chain for temperature specific drugs. The Raft consensus algorithm has been used to improve the throughput of the system and Blockchain Distributed Network (BDN) with bloXroute servers has been used to improve the network scalability. To provide privacy and security to the system, we introduced a novel hybrid approach of several cryptographic techniques. However, the framework is more flexible with the requirements of the system and several components can be changed or removed. By solving the network bottleneck issue using bloXroute, other consensus algorithms with higher fault tolerance can also be adjusted in the proposed model. Finally, we show full implementation to deploy the system and conduct rigorous performance evaluation and detailed security analysis of a scalable blockchain network. 

## Figures and Tables

**Table 1:** Comparison of the most popular blockchain consensus protocols.

Property	PoW [19]	PoS [27]	Raft [28]
Blockchain type	Public	Public/Private	Permissioned
Network Scale	Large	Large	Small
Throughput	Small	Small	High
Security	High	Little High	General
Transaction confirmation time	Long	Medium	Short
Application	Bitcoin, Ethereum	Peercoin	Hyperledger Fabric
Energy consumption	High	Medium	Low
Fork	Easy	Easy	Not possible

**Table 2:** Security level of cryptosystem with several key lengths.

Algorithm Family	Cryptosystem	Security Level			
		80	128	192	256
Elliptical Curve	ECDSA, ECDH	160 bit	256 bit	384 bit	512 bit
Integer factorization	RSA	1024 bit	3072 bit	7680 bit	15,360 bit
Discrete logarithm	Elgamal, DSA, DH	1024 bit	3072 bit	7680 bit	15,360 bit

**Table 3:** Battery drain (mAh) for IoT devices.

Cipher	Basic	Advanced
AES	2.52	2.82
SPECK	1.54	1.68
LEA	1.60	1.81

**Table 4:** Evaluation of security requirements.

Requirement	Model Solution
Authorization	Public key and digital ring signature
Data integrity	Hashing the data blocks
Data Confidentiality	Lighweight encryption
Network Scalability	BloXroute server
Throughput Scalability	Raft consensus algorithm
Privacy and Anonymity	Digital ring signature and Hyperledger
Quick response (QR) code security	Watermark based clone-proof QR code
